# Venomous and Poisonous Australian Animals of Veterinary Importance: A Rich Source of Novel Therapeutics

**DOI:** 10.1155/2014/671041

**Published:** 2014-07-21

**Authors:** Margaret C. Hardy, Jonathon Cochrane, Rachel E. Allavena

**Affiliations:** ^1^Institute for Molecular Bioscience, The University of Queensland, St Lucia, QLD 4072, Australia; ^2^School of Veterinary Science, The University of Queensland, Gatton, QLD 4343, Australia

## Abstract

Envenomation and poisoning by terrestrial animals (both vertebrate and invertebrate) are a significant economic problem and health risk for domestic animals in Australia. Australian snakes are some of the most venomous animals in the world and bees, wasps, ants, paralysis ticks, and cane toads are also present as part of the venomous and poisonous fauna. The diagnosis and treatment of envenomation or poisoning in animals is a challenge and can be a traumatic and expensive process for owners. Despite the potency of Australian venoms, there is potential for novel veterinary therapeutics to be modeled on venom toxins, as has been the case with human pharmaceuticals. A comprehensive overview of envenomation and poisoning signs in livestock and companion animals is provided and related to the potential for venom toxins to act as therapeutics.

## 1. Introduction

Australia is justifiably famous as the island continent with the most venomous and poisonous animals. These include native animals like Australian venomous snakes and introduced species like the cane toad. Many of these species pose a significant health risk to companion animals and livestock and thus are of both veterinary and economic importance.

Animal venoms are used effectively for defense and predation; poisons are used primarily for protection from predation. Both venoms and poisons are complicated cocktails, consisting of several hundred different components. Venom toxins are the primary actors for toxicity in animal venoms, particularly for invertebrate venoms [1]. Venom toxins are peptides, generally 3–6 kDa in size containing between 2 and 4 disulfide bonds, in a highly stable inhibitor cystine knot (ICK) motif [2]. ICK venom toxins can have a wide range of activities, including ion channel blockers (including neurotoxins), hemolytic agents, and antiviral or antibacterial agents. Toxins are distinct from enzymes, larger proteins, and nonpeptidic components like alkaloids and polyamines, and toxins are responsible for much of the biological activity and pharmacological interest around animal venoms and poisons.

Australia's most dangerous venomous snakes are front-fanged elapids and their venoms are potent and diverse. Further, they are common in both rural and urban areas posing a significant health risk to domestic companion animals and livestock. Snake venoms primarily contain procoagulants, anticoagulants, neurotoxins, myotoxins, and nephrotoxins; however, the locally acting necrotoxins generally found in non-Australian elapid and viper venoms are largely absent [3].

Cane toads are introduced amphibians that have been wreaking havoc on Australian ecosystems since their introduction in 1935 [4]. The cane toad has a highly toxic paratoid secretion that is particularly toxic to dogs [5]. Cane toad poison is composed primarily of biogenic amines, bufadienolides, alkaloids, and peptides and proteins [6]. Ontogenic variation in the cane toad poison has been reported, and the eggs contain higher concentrations and a wider range of active compounds than do adult toads [7]. The poison in the parotid glands induces neurologic or respiratory signs in dogs and cats when the toads are mouthed or ingested, and effects of poisoning can be so severe that death results despite treatment [8].

The Australian paralysis tick,* Ixodes holocyclus* (Acari: Ixodidae), contains toxins, particularly holocyclotoxin, in its saliva which can be lethal to companion animals and livestock [9]; an antidote is available for paralysis ticks. For other invertebrate species, anaphylaxis or localized severe reactions are the primary concern for their bites and stings [10]. Insects cause clinical signs related to bites and stings, may cause anaphylaxis, and may be poisonous if ingested in the case of sawfly larvae or caterpillar species with urticating hairs or spines [11]. Australian tarantulas (Araneae: Theraphosidae) are unique in that they have been shown to be lethal to canids, but not to humans [12]. Scorpions are of clinical importance because of their neurotoxic venom, which affects both humans and animals [13], and no scorpion antivenom currently exists.

The diverse range of pathophysiological effects of the venoms and toxins from Australian venomous and poisonous animals present a major challenge for veterinary treatment. Further, for many of Australia's venomous and poisonous animals no antivenom is available, and the clinical signs can only be treated symptomatically (including spider bites and cane toad poisoning). Venom and poison toxins can be a source of novel pharmaceutical agents, which is only recently being explored in humans [14]. The goal of this review is to provide an overview of venom and poison pathogenesis of veterinary import in Australia and discuss the potential for targeted compounds in drug discovery for animal therapeutics.

## 2. Venom Pathogenesis and Poisoning in Australia

### 2.1. Snakebite

Snake envenomation is an important presenting problem at veterinary clinics, with previous studies estimating the prevalence at 0.31% of clinical cases [15]. Another survey estimated up to 6,200 cases reported per annum, predominantly in dogs and cats, with 78% of cases occurring in rural versus 22% in urban areas [16]. Identifying the snake correctly is difficult in veterinary circumstances, given that the animal may be bitten in isolation (or while unsupervised) and the snake may not be presented with the animal for correct identification. A commercially available rapid freeze-dried sandwich enzyme immunoassay, the CSL snake venom detection kit (CSL Limited, Parkville, Victoria), is available for use in Australian animals. With significant treatment associated costs for hospitalization, often with intensive care and antivenom, most owners are reluctant to pay for the additional cost of a venom detection kit. In the late 1990s, the kit was estimated to be used in only 1% of cases [16]. If a snake venom detection kit is used, it is important to select the most appropriate test: a blood test, a urine test, a swab of the bite site, or a combination of all three.

A study of rapid immunoassay snake venom detection kits in an experimental model of tiger and brown snake envenomation in cats demonstrated that if envenomation occurred less than 8 hours previously, blood was the best sample; however, after 8 hours it was essential that urine be sampled [17]. Notably, a horse envenomated by a tiger snake gave a negative result from a serum sample venom detection kit (SVDK) but was strongly positive when a urine sample was used [18]. Although bite site swabs can be used, bite sites are rarely identified in animals either in life or during a postmortem examination. False positives with SVDKs have been anecdotally reported; however, a study on urine from 50 dogs and 25 cats presenting to veterinary clinics demonstrated no false positive reactions, so test specificity was estimated at 100% on urine as a test sample [19]. False negatives can occur with high venom concentration saturating binding antibodies in the kit (known as the “hook effect”), with venom levels below the limit of detection in subclinical envenomation, and insufficient time for venom to concentrate in the urine, or an extended period of time between envenomation and testing, which results in venom levels in urine below the level of detection [19].

**Figure 1:**
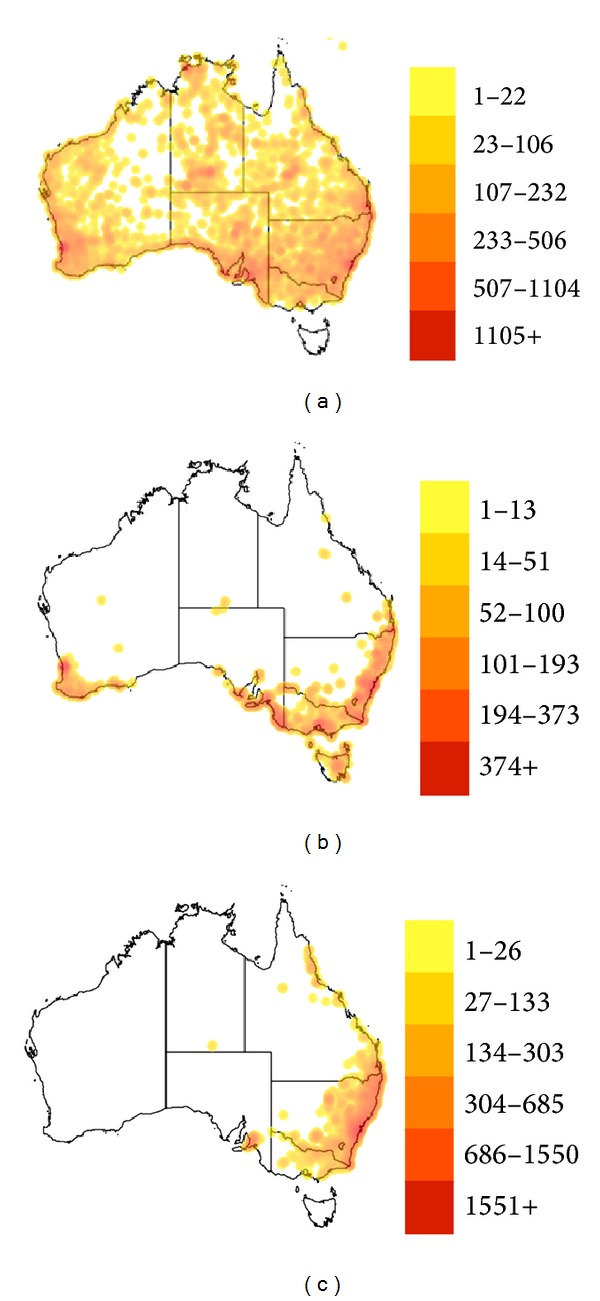
A map of the distribution of the three most commonly encountered Australian snakes of veterinary importance: the venomous brown snake (*Pseudonaja* spp., 11,923 records, (a)), the tiger snake (*Notechis scutatus*, 2,366 records, (b)), and the red-bellied black snake (*Pseudechis porphyriacus*, 4,017 records, (c)). Relative density is indicated by the legend to the right of each map. Maps from [20–22].

The three most commonly encountered snakes causing envenomation of veterinary importance are the venomous brown snake, the tiger snake, and the red-bellied black snake. The latter two snakes are mostly localized near the coast, particularly the east coast, but the brown snake is ubiquitous throughout the continent; the tiger snake is the only one recorded in Tasmania (Figure 1).

#### 2.1.1. Venomous Brown Snakes (*Pseudonaja* spp., Elapidae)

Venomous brown snakes in the genus* Pseudonaja* are distinct from unrelated brown snakes whose habitats overlap, including the venomous king brown (*Pseudechis australis*, from the black snake genus) and the taipan (*Oxyuranus scutellatus*) and nonvenomous brown-colored snakes like pythons. Brown snake envenomation is characterized by a severe lower motor neuron paralysis with hypocoagulation [23]. Animals suffer an initial haemodynamic collapse with severe systemic hypotension and thrombocytopenia [23, 24]. In an experimental model using anaesthetized dogs hemodynamic effects of brown snake (*Pseudonaja* spp.) venom included hypotension with reduced cardiac output and stroke volume and a rise in peripheral vascular resistance and a transient increase and then decrease in heart rate [25]. Hematological effects consistent with significant derangement of coagulation included marked thrombocytopenia, depletion of serum fibrinogen, prolonged prothrombin, and activated partial thromboplastin time [24]. The group C prothrombin activators in brown snake venom closely resemble mammalian prothrombinase (Xa:Va) which converts prothrombin into thrombin; thus the venom activates coagulation resulting in a consumptive coagulopathy termed venominduced consumptive coagulopathy [26].

*Pseudonaja* venom also contains several neurotoxins: a potent presynaptic neurotoxin (textilotoxin) and two postsynaptic peptidic neurotoxins (pseudonajatoxin) [27]. The clinical signs resulting from these toxins appear to be highly variable amongst envenomated species. Humans rarely demonstrate neurotoxicity of clinical significance (“the brown snake paradox”) [27], whilst ascending flaccid paralysis and respiratory muscle failure are a much more common finding in dogs and cats [15].

#### 2.1.2. Tiger Snake (*Notechis scutatus*, Elapidae)

Tiger snake venom contains a number of neurotoxins, procoagulant factors, and a weak haemolysin, resulting in a primarily neurological, myolytic and coagulopathic clinical syndrome [18, 28]. The complex presentation of tiger snake envenomation has been classified into three categories of clinical signs: (1) a preparalytic phase (acute collapse, vomiting, hypersalivation, defecation, trembling, and tachypnea), a paralytic and lethal phase (skeletal muscle paralysis, coagulopathy, and oliguria, with or without myoglobinuria or haemoglobinuria), and a sublethal or delayed phase (mydriasis, reduced pupillary light reflex, stiffness, ataxia, inability to close the jaw, and/or renal failure) [28]. During the preparalytic stage collapse, vomiting, salivation, defecation, trembling, and tachypnea are observed. Skeletal muscle paralysis, coagulopathy, and oliguria (which may include either myoglobinuria or haemoglobinuria) are noted in the paralytic stage and dilated pupils with absent pupillary light reflex, stiffness, and ataxia, inability to close the jaws, and renal failure are noted in the sublethal phase.

The principle neurotoxin, notexin, is a toxic phospholipase A_2_ that depletes acetylcholine [18]. Notexin is also a potent myotoxin and can cause extensive skeletal muscle degeneration, though with rapid death insufficient time may elapse for significant skeletal muscle changes to occur [18, 29]. A procoagulant with factor Xa-like activity is present and histopathological studies on a dog and cat which died for tiger snake envenomation demonstrated extensive thrombus formation [18, 29].

Clinical features of a horse diagnosed with tiger snake envenomation by sandwich ELISA included muscle fasciculation, reluctance to move, profuse sweating, tachycardia, tachypnea, and localized hot painful swelling on the muzzle presumed to be the bite site though punctures were not visible [18]. Significant hematologic abnormalities in this horse included mild neutrophilia with a left shift but no toxic changes and mild elevations in fibrinogen. For clinical chemistry, the horse exhibited a range of hematologic abnormalities with the most notable being increased creatinine kinase and aspartate aminotransferase likely due to muscle damage, and the animal had a significant myoglobinuria.

#### 2.1.3. Red-Bellied Black Snake (*Pseudechis porphyriacus*, Elapidae)

Red-bellied black snake venom is reported to be strongly haemolytic and weakly neurotoxic; however few reports of envenomation by* Pc. porphyriacus* in domestic animals are present in the literature [23]. Envenomation by* Pc. porphyriacus* has been reported to cause intravascular hemolytic anemia, rhabdomyolysis, and anuric renal failure secondary to myohemoglobinuric pigmenturia in a dog [30]. In humans,* Pc. porphyriacus* envenomation causes necrosis around the bite site, pigmenturia, increased serum creatinine kinase, and systemic signs like sweating, nausea, and headache [31].

### 2.2. Poisoning by Cane Toads (*Bufo marinus*, Anura: Bufonidae)

**Figure 2:**
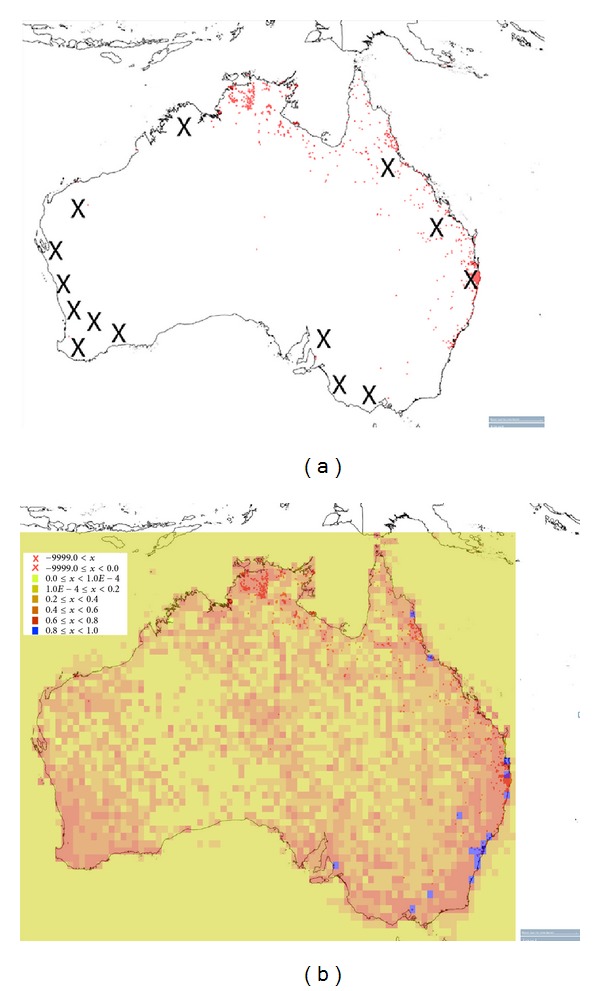
A map of the current cane toad distribution (6,349 total records marked with red dots (a)) and 14 of the 15 biodiversity hotspots (×, (a)). The cane toad population data is overlaid with species richness data; blue indicates higher and yellow lower levels of species diversity, respectively (b). Maps created from [33].

Cane toads have been an invasive pest in Australia for nearly 80 years and in that time have decimated native animal populations and destroyed pristine habitat [4, 32]. The Australian Government Department of the Environment has identified 15 biodiversity hotspots in Australia (Figure 2(a)); the cane toad is already in five of those locations and has the potential to invade at least three more. The 15th biodiversity hotspot is in Tasmania, where no cane toads have been recorded. To give a clearer picture of the danger cane toads pose to native Australian fauna, the level of species richness has been overlaid with the cane toad population map (Figure 2(b)).

Cane toad poison induces neurological and cardiovascular effects and exposure to cane toad poison can be lethal to both dogs and cats [5, 8]. The poisonous skin of cane toads (*Rhinella* =* Bufo marinus*, Anura: Bufonidae) contains high concentrations of orally active compounds and is the main reason their toxicity in predatory animals is so high. Contaminated drinking water and food is a particularly insidious exposure route and good hygiene can go a long way towards reducing that risk for pet and livestock caretakers. Cane toad poison consists in large part of bufadienolides, a steroid that is a type of cardiac glycoside. Interestingly, compared to other life stages, cane toad eggs contain both the highest number of individual bufadienolides and the highest concentration of those compounds compared to later-stage juveniles [7]. These compounds act by inhibiting the sodium-potassium pump and increasing the force of contraction by the heart, thus increasing cardiac output.

Cane toad poisoning is not just an Australian problem. In the United States, dogs and cats in Florida, Colorado, Arizona, Texas, and Hawaii have reported intoxication from contact with* Bufo* toads:* B. marinus*, the cane toad, and* B. alvarius*, the Colorado river toad [34]. Dogs are more commonly poisoned than cats and terriers are disproportionately represented in the demographics [8, 34].

Exposure to cane toad poison produces some or all of the following signs: in America, neurological abnormalities, hyperemic mucous membranes, ptyalism, recumbency or collapse, tachypnea, and vomiting [34]; in Australia, ptyalism, hyperemic mucous membranes, and seizures [8]. Electrocardiographic findings were most commonly sinus arrhythmia, sinus tachycardia, and normal sinus rhythm [34]. The treatment for animals exposed to cane toad poison is lavage of the mouth and affected areas with tap water and the survival rate for the studies in both America and Australia discussed above was >90%.

### 2.3. Arthropods: Stings, Bites, and Poisoning

#### 2.3.1. Hymenoptera

The insect order Hymenoptera includes the Apoidea (bees), Formicidae (ants), Vespoidea (wasps, hornets, and yellow jackets), and Symphyta (sawflies). Bees lose their stinger after stinging and die, but vespids can sting multiple times and also bite. Ants bite and some secrete venom that travels through the wound created at the bite site. Venoms from the Apoidea and Vespoidea are primarily made of proteins, but formicid venoms are 95% alkaloids [35]. Although anaphylaxis due to rapid hypersensitivity is the primary concern with Hymenoptera venom [10], ant bites and stings have long been known to cause severe pain and irritation [36]. The estimated lethal dose is 20 stings/kg in most mammals, though anaphylactic reactions are not dose-dependent [35]. No antivenom is available for bites and stings by Hymenoptera; in most cases, management of clinical signs (including anaphylaxis) is the only recourse. This can generally be achieved through administration of fluids, corticosteroids, and supportive care [37].

Recently, the first account of survival after bumblebee-sting induced anaphylaxis in a dog was reported: “Over the following 48 hours, the dog developed azotemia, severely elevated liver enzyme levels, hypertension, hematochezia, hematemesis, and disseminated intravascular coagulation. The dog's neurologic status improved slowly, but significant behavioral abnormalities remained. The dog was discharged after seven days with ongoing polyuria, polydipsia, and behavioral changes. The polydipsia and polyuria resolved within a few days, but the behavioral changes continued for six weeks” [38]. In another case, a dog presented for respiratory stress and shock after being stung by >100 bees; acute lung injury/acute respiratory syndrome was diagnosed and after eight days of treatment with oxygen, steroids, antibiotics, and bronchodilators, the dog recovered [39].

Although not currently present in Australia, Africanized bee stings present a significant threat of veterinary concern should they colonize. A retrospective study of dogs envenomated by Africanized bees in Brazil demonstrated dark-colored kidneys, dark red urine, dark red lungs, and splenomegaly as the major gross changes [40]. Secondary to massive Africanized bee envenomation (stings by >300 Africanized bees) in a dog, immune-mediated thrombocytopenia was identified [41]. After a red blood cell transfusion, immunosuppressive dexamethasone, and gastroprotectant therapy, the dog stabilized and platelet count returned to normal within a week. In another case of bee sting envenomation, immune-mediated hemolytic anemia developed in two dogs; one dog died and the hemolysis in the other was resolved following prolonged administration of corticosteroids [42].

Sawfly poisoning in Australia is largely due to* Lophotoma *spp. and the major toxin that causes poisoning is lophyrotomin, an octapeptide that acts principally on the liver [43]. The intraperitoneal LD_50_ in mice for lophyrotomin is 2 mg/kg [44]. Livestock, particularly sheep and cattle, are exposed to sawfly poisoning when leaves on the ground have sawflies on them and are ingested [37]. After removing animals from the sawfly source, the recommended management of poisoning consists of administration of silymarin and penicillin and glucose to prevent toxicosis and significant changes to liver enzymes [37].

#### 2.3.2. Lepidoptera

In addition to the Hymenoptera, caterpillars of many Lepidoptera (butterflies and moths) contain urticating hairs and spines. In the early 2000s in the United States, eastern tent caterpillars (*Malacosoma americanum*, Lepidoptera: Lasiocampidae) were found to be responsible for mare reproductive loss syndrome (MRLS). The combined losses from 2001 to 2002 for the thoroughbred industry due to MRLS were estimated at $500 million and more than 4500 equine pregnancies (3,500 of those, or 17%, were from thoroughbreds) were lost [45]. In Australia, similar incidences of MRLS were reported in the mid-2000s, with* Ochrogaster lunifer* (Lepidoptera: Thaumetopoeidae) found responsible [46]. After experimental gavage caterpillar setal fragments were found in multiple organs including the liver and gastrointestinal and reproductive tract and caused serositis, ulceration, and inflammation and it was theorized that the setae could vector bacteria resulting in secondary bacterial abortion [46].

#### 2.3.3. Spiders

Australian spiders are notorious for being venomous and deadly. The Australian funnel-web spider is one of a handful of spiders worldwide that are lethal to humans and a bite from the redback spider causes latrodectism (hallmarks of which include pain, muscle rigidity, vomiting, and sweating) [47]. In addition to having dangerous or lethal effects in humans, animals also experience severe, and sometimes fatal, effects of envenomation.

**Figure 3:**
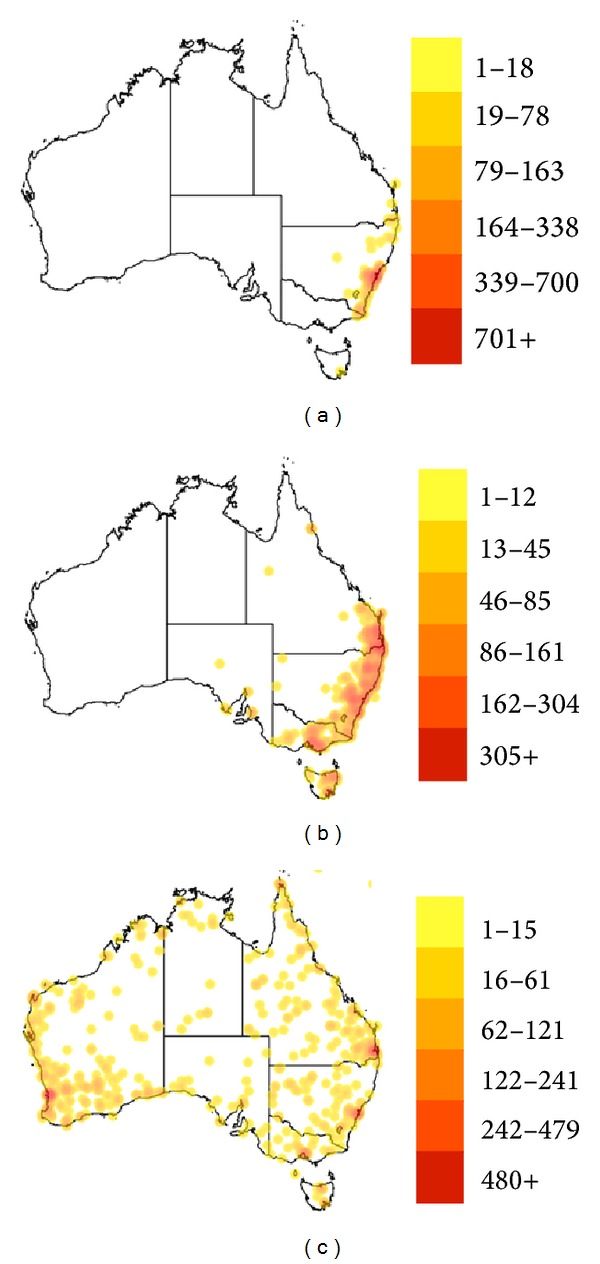
Density and distribution of the three most dangerous spiders in Australia. The Australian funnel-web spiders in the genera* Atrax* (1,526 records (a)) and* Hadronyche* (2,108 records (b)) are localized primarily on the east coast and the redback spider (*Latrodectus hasselti*) is more widely distributed (1,297 records, (c)). Maps from [48–50].

The distribution of dangerous Australian spiders varies. Australian funnel-web spiders are found primarily on the east coast, which is where the bulk of the human population has settled (Figures 3(a) and 3(b)). The redback spider, on the other hand, is widely distributed around the coastal areas and throughout the center of the country (Figure 3(c)). Unlike snakes, all three spiders have been found on the island of Tasmania.

The Australian funnel-web spider is classified into 35 species found in three genera,* Hadronyche, Illawarra*, and* Atrax* (Araneae: Hexathelidae) [53]. The lethal toxin in funnel-web spider venom, *δ*-HXTX-Ar1a, is a 4.8 kDa peptide with three disulfide bonds that was first described in 1985 [54]. Although the toxin is found in both males and females, only males seem to produce enough toxin to cause lethal effects after an envenomation [55]. The venom of the funnel-web spider has a wide phylogenetic range: rats, rabbits, and cats seem to be unaffected by a bite from a female spider, whereas 20% of mice and guinea pigs died after a bite from a female and most died after a bite from a male [37]. Male funnel-web spider bites have also been shown to have transient effects in dogs and cats [37]. Antivenom was introduced in 1984, after which no human fatalities from* A. robustus* or related spiders have been reported [56]. The LD_50_ of *δ*-HXTX-Ar1a has been reported as 0.16 mg/kg (33 pmol/g) in mice.

The redback spider,* Latrodectus hasselti* (Araneae: Theridiidae), is an Australian widow spider in the same genus as the North American black widow (*L. mactans*) and the New Zealand katipo (*L. katipo*). The major toxicity in animals is caused by *α*-latrotoxin-Lh1a, a 130 kDa presynaptic neurotoxin that causes the exhaustive release of neurotransmitters from presynaptic nerve terminals [57]. The reported LD_50_ value for* L. tredecimguttatus* (the European black widow) crude venom in guinea pigs is 0.0075 mg/kg in guinea pigs and 0.9 mg/kg in mice [37]. Although not naturally aggressive spiders, redbacks are widely distributed and accidental contact with humans and domestic animals can occur. Cats are particularly sensitive to* Latrodectus* venom; studies have reported an average survival time of 115 h and that 20 of 22 cats died after widow spider bites [37]. In humans, a bite of the Australian redback spider* Latrodectus hasselti* (Araneae: Theridiidae) causes latrodectism involving incapacitation through severe local, regional, or systemic pain and autonomic effects such as muscle rigidity and fasciculation, vomiting, dyspnoea, tachycardia, hypertension, weakness, and sweating [13]. Antivenom is available, but treatment is largely focused on symptom management [37].

**Figure 4:**
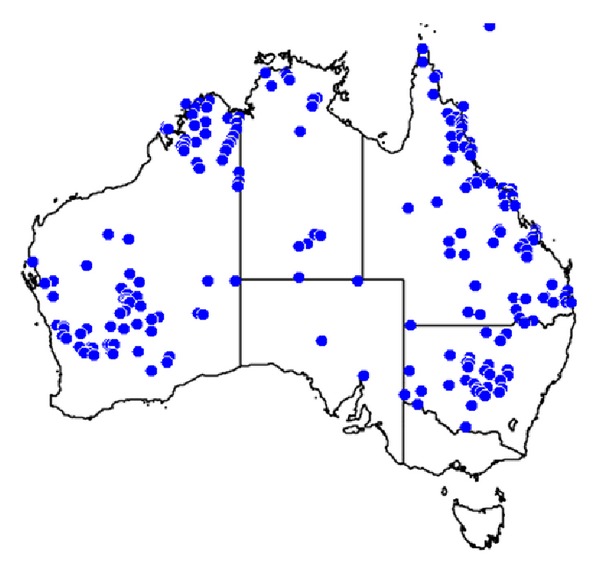
A distribution map of tarantulas (spiders in the family Theraphosidae), showing 463 occurrence records each marked with a blue dot. Map from [51].

Australian tarantulas or whistling spiders (Araneae: Theraphosidae) are extraordinarily lethal to companion animals. Dogs have been reported to be especially sensitive to tarantula envenomation and death is reported to occur in 30–120 min for most dogs [12, 58].* Phlogiellus *and* Selenocosmia* genus spider bites were reported to kill 7 dogs often within 2 hours of envenomation with apnea and cardiac arrhythmia as clinical features [12]. Australian tarantulas belong to four genera:* Selenotholus*,* Selenotypus, Coremiocnemis, *and* Phlogius*. Tarantulas are widely distributed throughout the Australian continent and North Queensland has a high concentration of tarantulas and people, which is why many cases of dog death are reported from that region (Figure 4).

Tarantula venom contains a variety of peptides with different mechanisms of action and venoms can be expected to contain neurotoxins, as well as possibly cytotoxic and hemolytic toxins. Following a tarantula bite, patients may experience muscle spasms, edema, hemoglobinuria, jaundice, and circulatory shock [37].

The venom of one species of Australian tarantula,* Selenotypus plumipes*, has recently been the source of the most potent orally active insecticidal peptide reported from spider venom [59]. Tarantulas are large, heavy-bodied spiders that live for 5–10 years in laboratory environments and Australian tarantula venoms contain a particularly large concentration of peptides in the 3–6 kDa range, within the size range of many active toxins and pharmaceutical leads [60].

#### 2.3.4. Ticks

Ticks affect animal and human health globally and cause significant economic losses directly via feeding, indirectly through the transmission of tick-borne diseases, and through toxicosis, a toxic reaction due to a toxic component present in the saliva. Ticks in the genus* Ixodes *are well known for their ability to induce paralysis during and after feeding [61]. The toxicity of ticks, which are hematophagic ectoparasites, comes from antigens in their saliva that modulate the host's immune response in order to facilitate blood feeding. Tick salivary anticoagulants are reported to act through either the inhibition of thrombin or inhibition of factor X activation [62].* Ixodes *tick paralysis is a toxin-mediated type of acute flaccid paralysis caused by the presynaptic neurotoxin holocyclotoxin, which acts to inhibit acetylcholine release at the neuromuscular junction [63–65]. Death is commonly the result of respiratory failure from a combination of neuromuscular paralysis causing hypoventilation as well as pulmonary parenchymal disease, though unexpected or “sudden” death is also reported [65, 66]. Dogs with tick paralysis may exhibit pulmonary congestion and oedema in uncomplicated cases but frequently also show moderate to severe bronchopneumonia with or without evidence of aspiration [65]. Laryngeal and oesophageal dysfunction, often accompanied by vomiting, is common in tick paralysis and may predispose affected dogs to aspiration pneumonia [65]. Further, analysis of crude toxin in rats indicates that* Ixodes* toxins have direct cardiovascular effects suggestive of potassium channel blockade [67]. Necropsy findings in other tissues are nonspecific and include severe vascular congestion in the liver, kidneys, and myocardium [68]. The first example of immunization against the paralyzing effects of holocyclotoxin was in dogs, using salivary gland extracts from* I. holocyclus*; after immunization, dogs were able to withstand four times the ED_50_ [69]. Australian paralysis ticks have a reported ED_50_ of 0.48 mg salivary gland protein/kg bodyweight to cause hind limb paralysis in dogs [69]. Despite the potency of salivary gland extracts, the amount of crude starting material extracted from ticks is extraordinarily small, which complicates discovery-stage work. Research suggests there may be different modes of action for toxins in the saliva of North American and Australian tick species [70].

**Figure 5:**
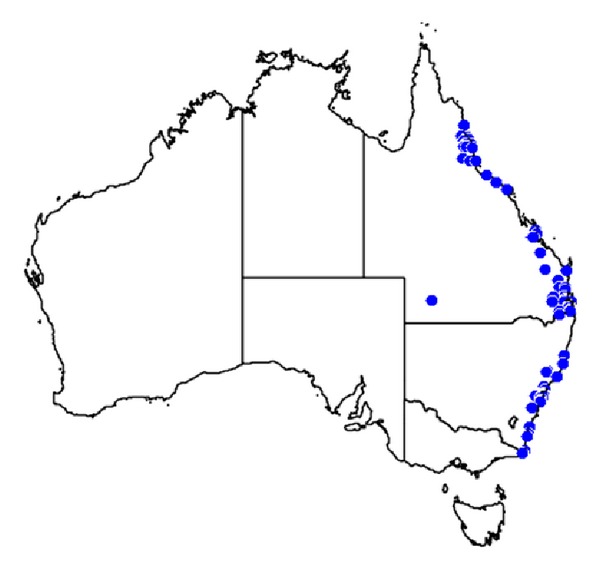
A distribution map of* Ixodes holocyclus*, the Australian paralysis tick. Each occurrence record (174 total) is marked with a blue dot. Map from [52].

The Australian paralysis tick is found primarily on the east coast (Figure 5).

Native hosts of* I. holocyclus* include the three species of bandicoot, although the tick has been found on a wide variety of native animals and livestock [71]. Although cats, dogs, and horses present most frequently with signs of tick infestation, paralysis ticks also affect native animals. The spectacled flying fox (*Pteropus conspicillatus*, Chiroptera: Pteropodidae) has shown affected electrical cardiac function when infested with* I. holocyclus* [72].

## 3. Potential for Novel Therapeutics

Recent technological advances have provided the gateway to exploring venomics (or, in the case of ticks, sialomics) as a novel source of therapeutics. First, the ability of proteomics and genomics to identify all the venom components, even those expressed in low quantities in the venom, allows more potent toxins to be identified. Second, the advent of high-throughput assays and target-based drug design have led to an explosion of interest in venom toxins, which can act as highly specific pharmacological probes for a single molecular target. Third, the bulk of vertebrate and invertebrate venom toxins hit ion channels, which are critical for nervous system function and an area of particular interest for pharmaceutical companies. Spider venom toxins, for example, represent one-third of known Na_V_ channel modulators [73].

Animal models of human disease are a critical component of drug discovery, although they can differ significantly from human biology and pathobiology [74]. Dogs are often a close match for human disorders, particularly for cardiovascular disease [75]. Thus, an understanding of the clinical effects and signs of envenomation in animals can yield pharmaceutical leads for veterinary use, as well as potential leads for human therapeutics, too.

### 3.1. Potency and Mechanism of Animal Venoms and Poisons

One of the advantages of using venom and poison toxins for pharmaceutical leads is the potency and highly targeted nature of the individual toxins. Two specific sources of potent potential veterinary leads are discussed further: snake venoms and cane toad poisons.

#### 3.1.1. Snake Venoms

A common thread between Australian elapid snake venoms is the presence of variations on potent *α*-neurotoxins [76]. The “*α*-” prefix is used to indicate toxins with postsynaptic activity; *α*-neurotoxins are neurotoxic peptides between 60 and 75 residues in length, which are linked by 4-5 disulfide bridges [77]. Short- and long-type toxins have similar 3D structures, but different dissociation kinetics with the receptor [77]. They act as competitive and irreversible antagonists of postsynaptic nicotinic acetylcholine receptors [78].

A variety of new human pharmaceuticals have been discovered from snake venoms, including several which are in clinical trials. Snake venoms have proved to be a particularly rich source of cardiovascular drugs [79]. Despite the potency of Australian snake venoms, their pharmaceutical use remains undetermined; to date, no human pharmaceuticals have been isolated from Australian snake venoms. Cenderitide, a toxin from the Eastern green mamba (*Dendroaspis angusticeps*, Squamata: Elapidae), is indicated in the treatment of congestive heart failure; a chemically modified version of a short-chain *α*-cobrotoxin, a cobra venom toxin (isolated from* Naja* spp. venom), is indicated in the treatment of HIV; and a chemically modified version of a long-chain *α*-cobrotoxin is indicated in the treatment of multiple sclerosis and perioperative bleeding [14]. Based on these examples of human pharmaceuticals, there is evidence to suggest novel therapeutics could be developed for veterinary use as well.

The chemistry of snake venoms has been fairly well characterized, primarily due to their importance in human medicine [80]. Snake venoms are produced in specialized venom glands and snake venom from an individual or within a species can vary widely [81], making treatment more of a challenge. Snake venom toxins are of particular interest for cardiovascular disease [79] and as natriuretic peptides, which modulate body fluid volume [82]. An overview of snake venom toxins is provided, with an emphasis on clinical effects (Table 1). Since these classes have major pathophysiological effects in snakebite victims, they are well suited to rational drug design.

#### 3.1.2. Cane Toad Poison

Several species of toad in the genus* Bufo* (Anura: Bufonidae) have been reported to have hallucinogenic or psychedelic effects when they are licked by humans. The bulk of these effects are due to the presence of an alkaloid, bufotenine, which is structurally related to the neurotransmitter serotonin, in skin secretions of the toad [83]. The hallucinogenic effects of licking cane toads have been reported in humans but not so comprehensively studied in dogs. Nonetheless, owners and veterinarians report poisoned dogs as appearing “high.” Not surprisingly, this Australian story has caught the public's interest at home [84] and overseas [85–87]. The behavior of these dogs belies some critical clues for the use of the poison extracts as potential therapeutics: (i) the poison components are orally active in dogs; (ii) the poison contains active compounds with neurological, cardiac, and potentially psychoactive effects specific to dogs; and (iii) after treatment, within 24 h of initial exposure the dog experiences complete recovery with no known long-term effects.

Cane toad poison consists in large part of bufadienolides, a steroid that is a type of cardiac glycoside. Interestingly, compared to other life stages, cane toad eggs contain both the highest number of individual bufadienolides and the highest concentration of those compounds compared to later-stage juveniles [7]. These compounds act by inhibiting the sodium-potassium pump and increasing the force of contraction by the heart, thus increasing cardiac output.

**Figure 6:**
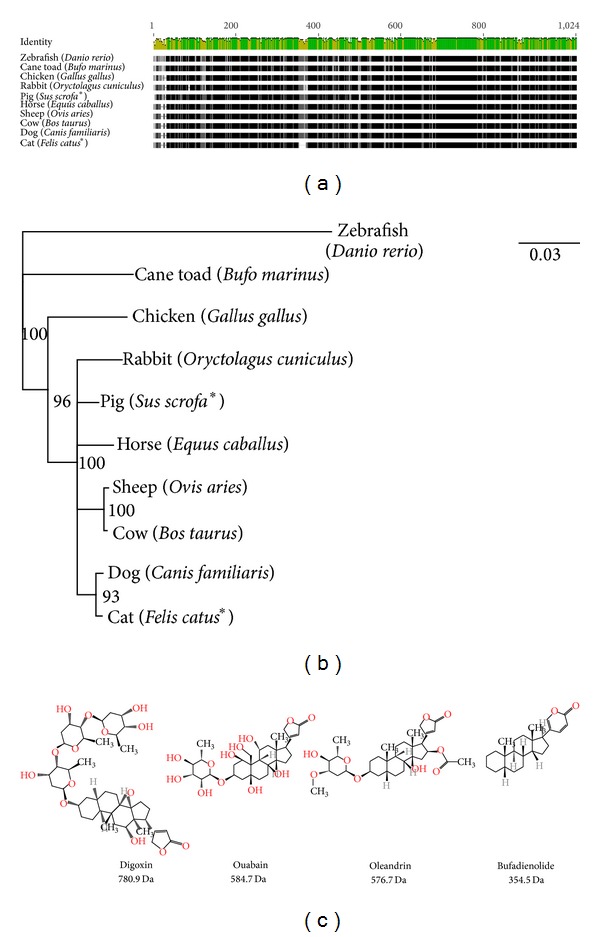
An alignment (a) and cladogram (b) of the ATP1A1 gene, which produces the sodium/potassium-transporting ATPase subunit alpha-1. A Jukes-Cantor genetic distance model of the ATP1A1 gene, using a neighbour-joining tree building method with zebrafish as the outgroup (b). Bootstrapping was used as a resampling method with 100 replicates and the support threshold was 50%. An asterisk (∗) in (a) and (b) indicates a partial sequence from UniProt. Chemical structures in (c) are from ChemSpider (http://www.chemspider.com/); the average mass is reported.

Despite the toxicity of cane toad poison to other vertebrates, including reptiles and mammals, cane toads and chickens are to be immune to the poison; in fact, one chicken was reported to eat 45 cane toads over a two-day experimental period with no ill effects [88]. The same study showed chickens had no adverse reaction when drinking water cane toads which had been sitting in overnight, suggesting perhaps chickens are nonresponsive to the cardiac glycosides in the cane toad poison. Chickens and cane toads have slightly, but not completely, different gene sequences for the sodium-potassium pump (Figures 6(a) and 6(b)). The gene sequences for cats and dogs are most similar to each other, and most different from chickens and cane toads. These gene-level differences may explain why companion animals (cats and dogs) are so susceptible to cane toad poisoning and livestock (rabbits, pigs, sheep, and cows) are protected from the severe cardiac effects of cane toad poisoning.

Cardiac glycosides are commonly prescribed to treat congestive heart failure and arrhythmia and several successful drugs have been developed from natural products (Figure 6(c)).

Not surprisingly, these naturally derived cardiac glycosides are considered lethal when encountered in nature; however, therapeutic doses can usually be achieved. Ouabain is an exception, as it is so potent that it is largely only used experimentally. As with the commercially available drugs, the reaction of dogs to cane toad poisoning is delayed. Currently, no specific antidote for cane toad poisoning exists and clinical management relies on lavage of the mouth and exposed areas to decrease toxin exposure, followed by symptomatic management. Bufadienolide is the smallest of the naturally derived cardiac glycosides and its synthesis was first demonstrated in the literature in the 1970s [89]. Bufadienolides also have demonstrated antitumor activity; derivatives of one alkaloid, bufalin, have been shown to have antiproliferation activity against carcinoma and leukemia cells [90].

## 4. Conclusions

Australia has a variety of venomous and poisonous animals that are dangerous to humans, pets, and livestock. Costs of treating a single envenomation event can run into several thousand dollars and, despite extensive medical treatment, many animals die. A greater understanding of individual toxins will enhance our ability to diagnose and treat envenomation and poisoning and to monitor for secondary toxic effects. Although pathophysiology and treatment can be extrapolated from human studies, species differences occur and those venoms that have different effects in animals may prove to be a rich source of novel, specific veterinary therapeutics. Elucidation of the pathophysiology and mechanism of action of venoms and toxins will allow the development of novel human and veterinary therapeutics through rational drug design.

## Figures and Tables

**Table 1:** An overview of the major toxin classes with clinical effects in snake venom and their indications. Note myotoxins are necrotic and often lead to death via diaphragmatic paralysis.

Toxin class	Representative toxin	Clinical effects
Cardiotoxin	Cardiotoxin III	Irregular or ceased heartbeat
Hemotoxin	Convulxin	Hemolysis or coagulation
Myotoxin	Crotamine	Muscle necrosis
Nephrotoxin	RVV-7	Decreased creatinine clearance
Neurotoxin		
Presynaptic	Dendrotoxin	Nerve paralysis
Postsynaptic	*α*-Bungarotoxin	Numbness, paralysis
Anticholinesterase	Fasciculin	Extended fasciculation
